# RNA activating-double stranded RNA targeting flt-1 promoter inhibits endothelial cell proliferation through soluble FLT-1 upregulation

**DOI:** 10.1371/journal.pone.0193590

**Published:** 2018-03-06

**Authors:** Susie Choi, Hironori Uehara, Yuanyuan Wu, Subrata Das, Xiaohui Zhang, Bonnie Archer, Lara Carroll, Balamurali Krishna Ambati

**Affiliations:** 1 John A Moran Eye Center, University of Utah, Salt Lake City, Utah, United States of America; 2 Patanjali Research Institute, Haridwar, India; Kyung Hee University, REPUBLIC OF KOREA

## Abstract

Short-activating RNA (saRNA), which targets gene promoters, has been shown to increase the target gene expression. In this study, we describe the use of an saRNA (Flt a-1) to target the flt-1 promoter, leading to upregulation of the soluble isoform of Flt-1 and inhibition of angiogenesis. We demonstrate that Flt a-1 increased sFlt-1 mRNA and protein levels, while reducing VEGF expression. This was associated with suppression of human umbilical vascular endothelial cell (HUVEC) proliferation and cell cycle arrest at the G_0_/G_1_ phase. HUVEC migration and tube formation were also suppressed by Flt a-1. An siRNA targeting Flt-1 blocked the effects of Flt a-1. Flt a-1 effects were not mediated via argonaute proteins. However, trichostatin A and 5’-deoxy-5’-(methylthio) adenosine inhibited Flt a-1 effects, indicating that histone acetylation and methylation are mechanistically involved in RNA activation of Flt-1. In conclusion, RNA activation of sFlt-1 can be used to inhibit angiogenesis.

## Introduction

Angiogenesis, an essential physiological process of new vessel formation, is also a significant feature of tumorigenesis and many ocular diseases such as age-related macular degeneration and diabetic retinopathy [1–4].

Numerous proteins, including several growth factors, modulate the formation and maintenance of vasculature. Vascular endothelial growth factor (VEGF) is an important mediator of angiogenesis and has been a major therapeutic target for angiogenesis related diseases [1, 5]. Soluble fms-like tyrosine kinase-1 (sFlt-1) is a splice variant of VEGF receptor 1 (VEGFR-1 or Flt-1), one of the cell-surface receptors to which VEGF binds. sFlt-1 includes an extracellular domain that binds VEGF, but lacks transmembrane and intracellular domains, thus functioning as a decoy to sequester VEGF and prevent initiation of intracellular signaling [6]. We previously demonstrated that sFlt-1 is essential for maintaining avascularity of the cornea. Experimental knockdown of corneal sFlt-1 using RNA interference (RNAi) or conditional genomic deletion increases free VEGF and induces vascularization in the mouse cornea [7]. Modulating sFlt-1 expression has shown therapeutic potential for angiogenic diseases [1–4]. For example, upregulation of sFlt-1 induced by morpholino injection suppressed neovascularization in mouse models of laser-induced choroidal neovascularization (CNV) and corneal suture injury [8, 9]. sFlt-1 also showed its anti-tumor effect in mice transplanted with breast adenocarcinoma or ovarian cancer cells [9, 10]. Interestingly, serum sFlt-1 levels are associated with the progression of neovascular age related macular degeneration (AMD) in human patients [11], and clinical trials of sFlt-1 delivery via intraocular AAV vector injection are showing promise for the treatment of neovascular AMD [12, 13].

Modulating gene expression using non-coding RNAs (ncRNAs) is an active field of therapeutic development [14]. Functional gene silencing has been well established using small ncRNAs such as microRNAs, small interfering RNAs (siRNA), and PIWI-interacting RNAs. However, understanding of the role of ncRNAs in gene activation is in its early stages [15–17]. Although RNA activation has been utilized to enhance expression of numerous genes including tumor suppressor genes, designing effective small activating RNAs (saRNAs) is not straightforward [18, 19]. RNA activation is highly sensitive to the location of the target sequence relative to the transcriptional start site [20–22]. Also, the effect of a given saRNA may vary depending on the cell type and epigenetic state of the chromatin [18].

This study is the first to examine the potential of RNA activation for inhibition of angiogenesis by enhancing sFlt-1 expression. We show that saRNA for sFlt-1 inhibits endothelial cell proliferation, migration and tube formation by increasing the mRNA and protein levels of sFlt-1, possibly through histone modifications.

## Materials and methods

### saRNA design and pre-designed siRNA

The human flt-1 promoter and transcription start sites were determined from NCBI sequences (Nucleotide ID: ENST00000282397) and Morishita, K et al. [23]. Human Flt-1 saRNA design was based on guidelines provided by Li et al and Portnoy et al. [18, 24] and include the following: Flt a-1 (target position, between -724 and -742 from the transcriptional start): GUGCAUCAAUGCGGCCGAATT and UUCGGCCGCAUUGAUGCACTT; Flt a-2 (-987 to -1005): GAGGAACAACGUGGAAUUATT and UAAUUCCACGUUGUUCCUCTT; Flt a-3 (-1805 to -1823): GAGCUGAUGGAGGACUAAATT and UUUAGUCCUCCAUCAGCUCTT. Pre-annealed double stranded RNA was purchased from Thermo Fisher (Waltham, MA). Single stranded RNAs were purchased from the University of Utah core facility. For annealing single stranded RNA, 30μL of each RNA (100μM) was mixed with 15μL of 5x annealing buffer (250mM Tris-HCl at pH 8.0 and 500mM NaCl). Annealing was performed by incubating for 1 min at 95°C, 1 min 70°C followed by a 1°C decrease in temperature every 80 sec until 37°C was reached. For the small interfering RNA (siRNA) experiment, pre-designed siRNA to human Flt-1 and negative control No.1 siRNA was purchased from ThermoFisher (Cat.No. s5289 and 4390843 respectively).

### Cell culture and double stranded RNA transfection by RNAiMax

Human umbilical vein endothelial cells (HUVEC) were obtained from Lonza (Portsmouth, NH) and maintained in EGM-2 medium (Lonza). Cells were plated at 10,000 cells/well on a 24-well plate or 50,000 cells/well on a 6-well plate. The cells were incubated for 6 hours to overnight and transfection was performed with Lipofectamine RNAiMAX Transfection Reagent (Thermo Fisher) following the manufacturer's protocol. For example, for one well of a 24-well plate, double stranded RNA and 1μL of RNAiMAX were diluted with 50μl of OPTI-MEM (Thermo Fisher) and, incubated for 10 minutes before adding to the well. After 6 hours incubation, medium was replaced with 500μL fresh culture medium.

### Cell proliferation assay with crystal violet staining and flow cytometry

HUVECs were transfected with Flt a-1 at the indicated concentration. After fixing cells with formaldehyde for 15 min and washing with H_2_O, the cells were stained with 0.5% (w/v) crystal violet/ 25% (v/v) methanol for 25 min. After washing the cells with H_2_O until no color was eluted, the cells were dried. Crystal violet was then eluted with 10% acetic acid and the OD600 was measured.

Cell cycle analysis was performed by flow cytometry. Two days after the transfection, the cells were harvested with trypsin/EDTA and nuclei were stained with Vybrant^™^ DyeCycle^™^ Violet Stain (ThermoFisher) following the manufacture’s protocol. Data was collected using BD FACSCanto-II analyzer in University of Utah flow cytometry core.

### Cell migration assay

Cells were seeded on a 24-well plate and transfected with blank (RNAiMAX only), 10nM of dsCON, or 10nM of Flt a-1. The monolayer of cells was scratched with a pipet tip, and cells were imaged at 0, 4, 8, 12, 24, and 48 hours post-scratch. Four independent areas per treatment group were used for analysis. The cell-free areas were measured using ImageJ (NIH).

### Tube formation assay

HUVEC cells were transfected with blank (RNAiMAX), 10nM dsCON, or 10nM Flt a-1. 24 hours later, cells were collected and plated at 20,000 or 40,000/well on a 48-well plate coated with Matrigel (BD Biosciences, San Jose CA) with complete medium. Images were taken at a 24-hour time point under microscope. Quantification of tube networks was performed using AngioTool (20,000 cells) (NCI) and ImageJ with the Angiogenesis Analyzer plugin (40,000 cells) (NIH) [25, 26]. We used more suitable software depending on the quality of the image and the presence of cell aggregations. AngioTool was used to trace tube networks in both cell densities, however, ImageJ was more efficient at distinguishing between tubes and cellular clumps as cell density increased, and was therefore used for the 40,000 cell analysis.

### Reverse transcription and quantitative real-time PCR

Total RNA extraction, cDNA synthesis and real time PCR were described previously. Briefly, total RNA was purified using an RNeasy mini kit (Qiagen, Valencia, CA) or PureLink RNA Mini Kit (ThermoFisher). After treating total RNA with DNase I (Sigma, St. Louis, MO), cDNA was synthesized using Omniscript RT kit (Qiagen). QuantiTect SYBR Green PCR Kit (Qiagen) was used for real time PCR. The data was analyzed with ΔΔCt method. CT: threshold cycle, ΔCT = CTsFLT-1 or mbFLT-1 –CT_GAPDH_, ΔΔCT = ΔCT_transfection_− ΔCT_No transfection_ [27].

### Western blot analysis

Cells were lysed and protein concentrations were determined with a BCA assay (Pierce, Waltham, MA). Equal amounts of protein were loaded onto polyacrylamide gels and transferred onto PVDF membranes. Westerns were performed using anti-VEGF Receptor 1 (ab9540, 1:500, Abcam, San Francisco, CA), anti-VEGF (sc507, Santa Cruz, Dallas, TX), or anti-beta-actin (Abcam) primary antibodies. Appropriate secondary antibodies were purchased from Thermo Fisher.

### Genomic DNA methylation assay

Three days after transfection with 20nM Flt a-1, genomic DNA from HUVECs was purified using QIAamp DNA Mini kit (Qiagen). 500ng of each genomic DNA sample was processed using EZ DNA Methylation kit (Zymo Research, Irvine, CA) following the manufacturer’s protocol. 150ng bisulfite treated genomic DNA was used for PCR. PCR primers were designed using Bisulfite Primer Seeker 12S (Zymo Research). Two sets of PCR primers were used for amplification of the human flt-1 promoter; -474bp to -745bp Forward: TTGTTTTTAGGAAGTAGAAGATTGAGGAAATGATTTGG, Reverse: AAAAATCCRATCCAAAAAAAACTAACC and -111bp to 399bp Forward: GGYGGAGTTTTAGTTTTGTTTTTTTTTTAG, Reverse: TCCTACCCCRACACCTCCTTCTAATAAC. 95°C for 2 minutes, 40 cycles of 95°C for 15 seconds, 60°C for 30 seconds, 68°C for 1 minute. The final extension was 68°C for 5 minutes. The PCR product was run on 1% agarose gel and purified using gel purification kit (Qiagen). The PCR products were cloned into E.coli using CloneJET PCR cloning kit (Thermo Fisher). 10 colonies from each group were picked and DNA was isolated from each colony using miniprep kit for sequencing (Qiagen).

### Chromatin immunoprecipitation assay with anti-Ago-1 and anti-Ago-2 antibody

Ago-1/Ago-2 binding analysis was performed with the Chromatin Immunoprecipitation (ChIP) Assay Kit (Millipore, Temecula, CA) following the manufacturer’s instructions with some modifications. 3x10^6^ HUVECs were plated to 15cm culture dish. After overnight incubation, Flt a-1 or dsCON was transfected with RNAiMax at 10nM. After 8 hours incubation, the cells were collected, pelleted, and treated with nuclear extraction buffer (5mM PIPES, 85mM KCl, 0.5% IGPAL CA-630, pH 8.0 adjusted by NaOH) for 10 minutes on ice. After adding SDS lysis buffer, genomic DNA was sheared using sonic dismembrator model 100 (Fisher Scientific, Hampton, NH). We used power = 7, 6 times for 10 second intervals. For immunoprecipitation, we used monoclonal anti-Ago1 antibody clone 4B8 (SAB4200084, Sigma) and anti-Ago2 antibody clone 9E8.2 (04–642, Millipore). The same antibodies were used for western blot. For flt-1 promoter detection, we used the following primers: agttgcaggagcagtttcacg and gagcgtgtccgtgtctttttc. For positive control, we used the input genomic DNA which corresponds to approximately 1/100 of the input for immunoprecipitation.

### Treatment with trichostatin A or 5’-deoxy-5’-(methylthio) adenosine

HUVECs were transfected with Flt a-1 or dsCON at 10nM. After 6 hours, medium was changed with fresh medium containing 100nM TSA (ApexBio, Houston, TX), 300uM dMTA (Sigma), or blank and cells were harvested the next day for western blot analysis using anti-VEGF Receptor 1 (ab9540, Abcam) and GAPDH (Santa Cruz).

## Results

### RNA activation with Flt a-1 increases sFlt-1 mRNA and protein levels

**Fig 1:**
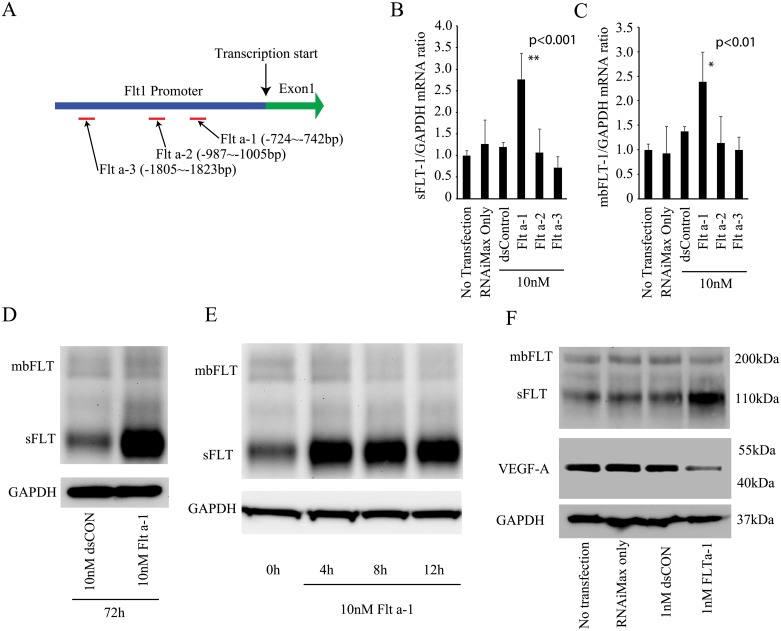
Flt a-1 enhances sFlt-1 expression at mRNA and protein levels. (A) Location of three different sFlt-1 saRNAs targeting the flt-1 promoter. (B,C) The construct Flt a-1, but neither Flt a-2, Flt a-3, nor controls significantly increased sFlt-1 and mbFlt-1 mRNA levels at 10nM. * and ** indicate p<0.01 and p<0.001 by Student’s t-test, respectively. Error bar is standard deviation. N = 6. (D) Flt a-1 enhanced sFlt-1 protein expression at 10nM. (E) The time-course experiment showing Flt a-1 induces sFlt-1 activation by 4 hours post-transfection. (F) Western blot showing 1nM of Flt a-1 can induce sFlt-1 activation. VEGF-1 levels decreased upon Flt a-1 transfection.

Three different 21 bp double stranded saRNAs (Flt a-1, Flt a-2 and Flt a-3) were designed and synthesized to target the human flt-1 promoter (Fig 1A) following previously reported protocols [18, 24]. To examine whether sFlt-1 saRNAs enhance sFlt-1 and membrane bound Flt-1 (mbFlt-1) expression, we transfected human umbilical vein endothelial cells (HUVECs) with each Flt-1 saRNA at 10nM. We found Flt a-1, but not Flt a-2 or Flt a-3, induced a significant increase in sFlt-1 and mbFlt-1 mRNA levels (Fig 1B and 1C). Next, we examined Flt-1 protein levels after Flt a-1 transfection by western blot. Seventy-two hours post Flt a-1 transfection, we found significant upregulation of sFlt-1 protein expression but not mbFlt-1 (Fig 1D). Surprisingly, 4h post transfection was sufficient time to detect upregulation of sFlt-1 (Fig 1E). We also found increased sFlt-1 protein levels with as little as 1nM Flt a-1 (Fig 1F). We measured sFlt-1 mRNA levels 72 hours after transfecting HUVECs with different concentrations (0, 1, 5, 10, 30nM) of Flt a-1 (S1 Fig). At 72 hours post-transfection, 5nM Flt 1-a treated group showed a significant sFlt-1 mRNA level increase similar to 10nm and 30nM, suggesting that the effect of 1nM Flt a-1 on sFlt-1 mRNA levels subsides over the 72 hour period, but we can still detect upregulation of sFlt-1 protein levels. Consistent with the upregulation of sFlt-1, we found simultaneous downregulation of endogenous VEGF-A protein (Fig 1F).

### Double stranded Flt a-1 suppresses endothelial cell proliferation

**Fig 2:**
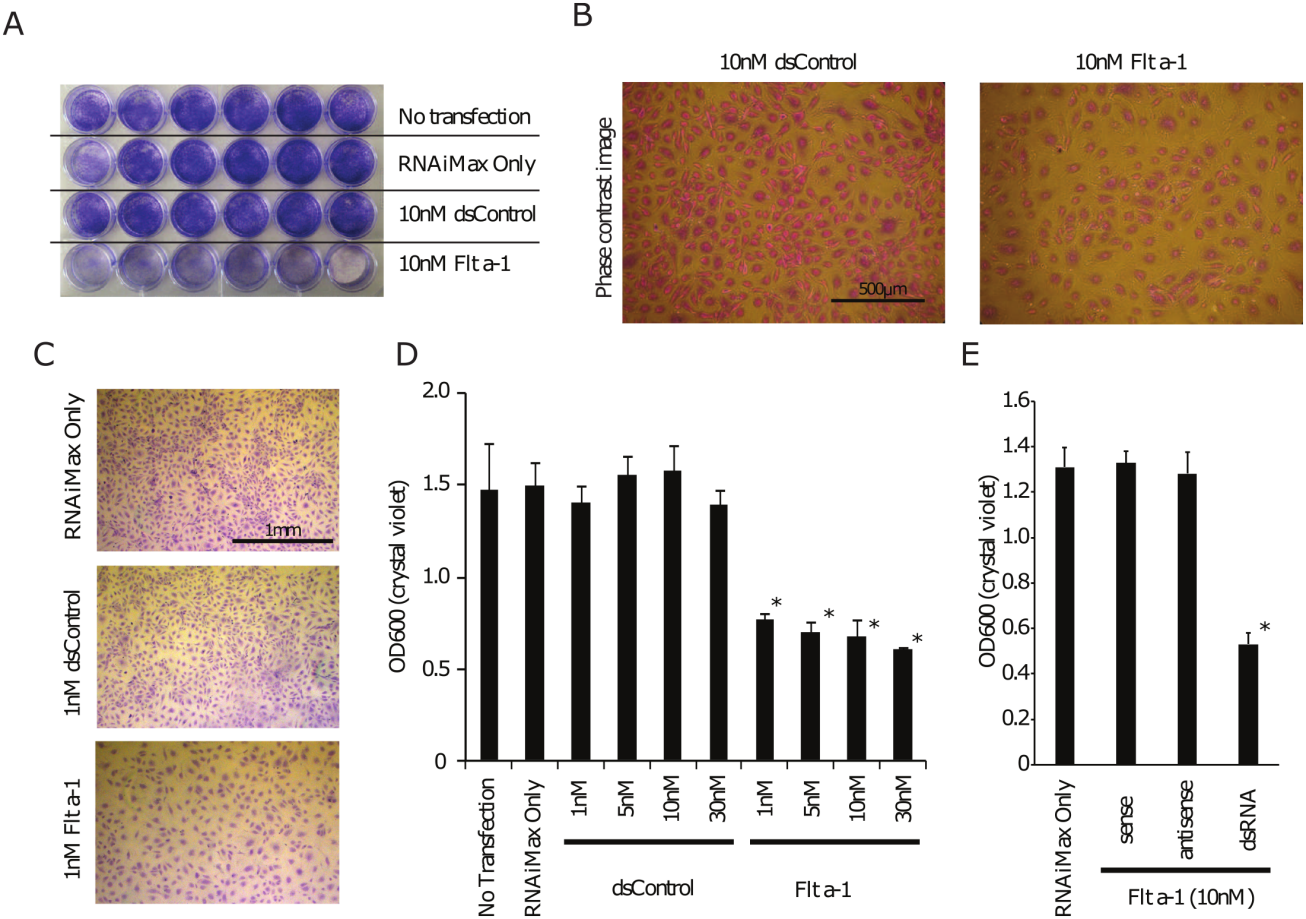
Flt a-1 inhibits endothelial cell proliferation. (A) HUVECs transfected with RNAiMax only, 10nM dsCON, or 10nM of Flt a-1 (N = 6) were fixed and stained with crystal violet at 72 hours post transfection. The Flt a-1 treated group showed obvious reduction of proliferation. (B) Phase contrast images of crystal violet-stained 10nM dsCON or 10nM Flt a-1 transfected cells. (C,D) Bright field microscope images and quantification of crystal violet staining confirm Flt a-1 induced inhibition of proliferation at 1nM. (E) Only double stranded Flt a-1, but not sense or antisense single stranded RNA alone, reduced proliferation of HUVECs.

Since Flt a-1 increases sFlt-1 expression and decreases endogenous VEGF-A protein levels, we examined whether Flt a-1 halts cell proliferation. Untreated HUVECs or HUVECs treated with RNAiMax alone, 10nM dsControl (dsCON), or 10nM Flt a-1 were cultured on 24 well plates and stained with crystal violet 72 hours after treatment (Fig 2A and 2B). Flt a-1 significantly inhibited proliferation with concentrations as low as 1nM (Fig 2C and 2D), consistent with levels sufficient for sFlt-1 protein upregulation (Fig 1F). While cells transfected with double stranded Flt a-1 showed decreased proliferation, neither sense, nor anti-sense single stranded Flt a-1 had any effect on proliferation (Fig 2E).

### Flt a-1 arrests the cell cycle mainly at G_0_/G_1_ phase

**Fig 3:**
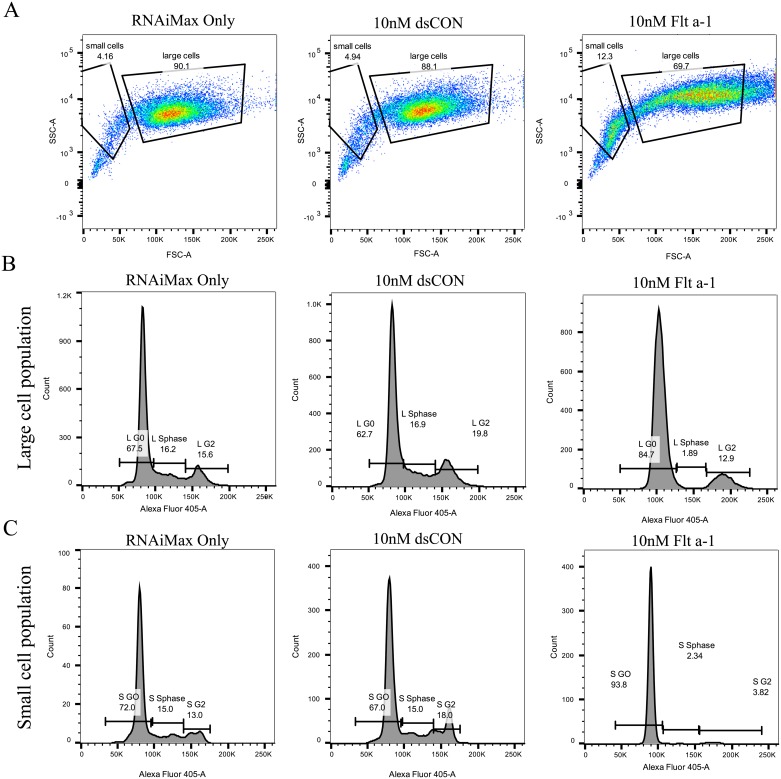
Flt a-1 arrests HUVEC cell cycle. (A) FSC vs SSC analysis showed two different cell populations. RNAiMax: small cells 4.16%, large cells 90.1%; 10nM dsCON: small cells 4.94%, large cells 88.1%; 10nM Flt a-1: small cells 12.3%, large cells 69.7%. (B) Cell cycle analysis of large cells. RNAiMax: G_0_ 67.5%, S phase 16.2%, G_2_ 15.6%; 10nM dsCON: G_0_ 62.7%, S phase 16.9%, G_2_ 19.8%; 10nM Flt a-1: G_0_ 84.7%, S phase 18.9%, G_2_ 12.9% (C) Cell cycle analysis of small cells. RNAiMax: G_0_ 72.0%, S phase 15.0%, G_2_ 13.0%; 10nM dsCON: G_0_ 67.0%, S phase 15.0%, G_2_ 18.0%; 10nM Flt a-1: G_0_ 93.8%, S phase 2.34%, G_2_ 3.82%.

Furthermore, we performed a cell cycle assay with flow cytometry. Since Side Scatter (SSC) vs Forward Scatter (FSC) indicated there are two populations of cells, we examined them separately (Fig 3A). In the large cell population (FSC high), the control HUVECs showed typical G_0_/G_1_, G_2_ and S-phase (Fig 3B, left and center). However, Flt a-1 treated HUVECs showed increased G_0_/G_1_ phase, decreased G_2_/M phase and diminished S-phase (Fig 3B, right). In the small cell population (FSC low), the control HUVECs showed the similar histogram to the large population (Fig 3C, left and center). Interestingly, the small population of Flt a-1 treated HUVECs showed only G_0_/G_1_ phase. Thus, Flt a-1 treatment stops the cell cycle at predominantly G_0_/G_1_ phase and partially G_2_/M phase.

### Flt a-1 impedes endothelial cell migration

**Fig 4:**
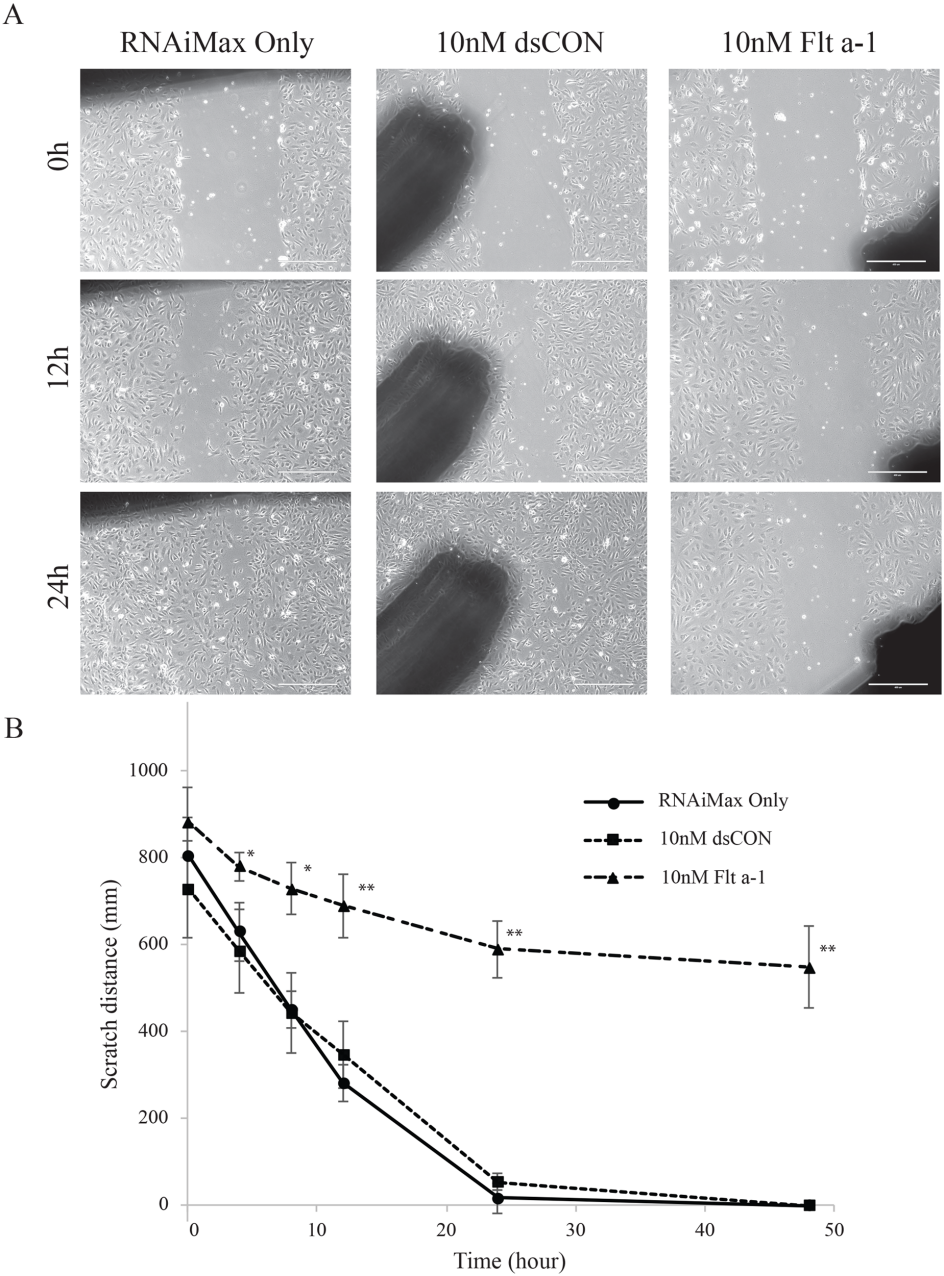
Flt a-1 impedes HUVEC migration. (A) Phase contrast microscope images at 0h, 12h and 24h after scratching. Scale bar is 400μm. The shadow on the pictures are locating marks. (B) Scratch distance on the time course (0h to 48h). * and ** indicate p<0.01 and p<0.001 compared to the controls by Student’s t-test. Error bar is standard deviation.

We evaluated the effect of Flt a-1 on HUVEC migration with an *in vitro* scratch assay. As shown in the representative images in Fig 4A, the cell-free scratch area in RNAiMax or DsCON transfected group was mostly closed at 24 hours post scratch, while it persisted in the Flt a-1 treated group. Quantification of 4 independent scratch areas per group confirmed that Flt a-1 reduced the migratory potential of HUVECs (Fig 4B).

### Flt a-1 inhibits endothelial cell tube formation

**Fig 5:**
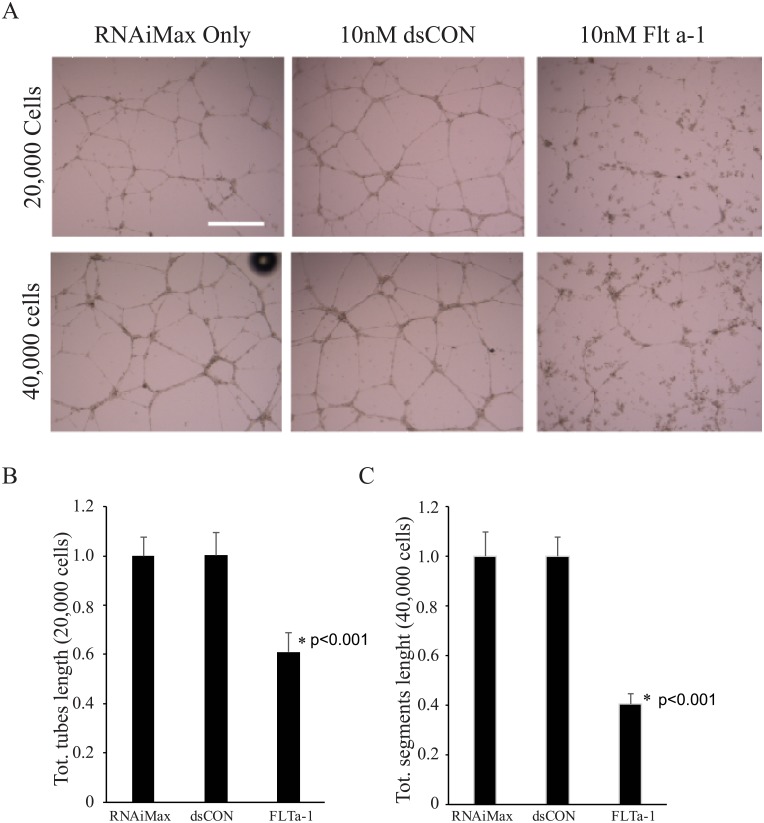
Flt a-1 inhibits in vitro tube formation. (A) One day after each treatment, 20,000 (top) or 40,000 (bottom) cells were plated on Matrigel on a 24 well plate. The representative images were shown after 24 hours incubation. N = 4. Scale bar is 500μm. Quantification of tube networks at 24 hours post-transfection when 20,000 cells (B) or 40,000 cells (C) were seeded. * indicate p<0.001 compared to RNAiMAX control by Student’s t-test. Error bar is standard deviation.

The effect of upregulation of sFlt-1 through Flt a-1 on HUVEC capillary-like structure (tube) formation was studied. HUVECs transfected with RNAiMax only, 10nM dsCON or 10nM Flta-1 were seeded on Matrigel^®^ and photographed after 24 hours (Fig 5). Cells treated with RNAiMax alone or dsCON formed a network of branching structures, while Flt a-1 transfected cells showed limited tube formation.

### Flt-1 siRNA rescues cell proliferation suppressed by Flt a-1

**Fig 6:**
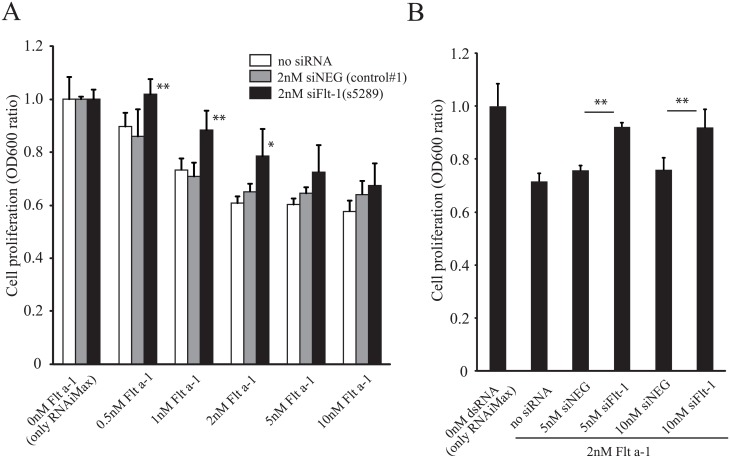
siFlt-1 rescues diminished HUVEC proliferation by Flt a-1. (A) HUVECs were transfected with RNAiMax only, 2nM siNEG or 2nM siFlt-1. After 6 hours incubation, different concentrations of Flt a-1 (0, 0.5, 1, 2, 5, 10nM) were transfected. After three days of incubation, the cell proliferation was analyzed by crystal violet. N = 4. (B) HUVECs were transfected with RNAiMax only, 5-10nM siNEG or 5-10nM siFlt-1, followed by 2nM Flt a-1. N = 4. * and ** indicate p<0.05 and p<0.01 by Student’s t-test. Error bar is standard deviation.

In order to test if reduced HUVEC proliferation after Flt a-1 transfection was specifically due to increased sFlt-1 expression, we knocked-down sFlt-1 expression with siRNA targeting Flt-1 (siFlt-1). HUVECs were transfected with 2nM siFlt-1, followed by transfection with different concentrations of Flt a-1 (0.5, 1, 2, 5, 10nM). We found that pre-treatment with siFlt-1 rescued the Flt a-1-mediated proliferation decline at lower concentrations of Flt a-1 (Fig 6A). We then tested whether higher concentrations of siFlt-1 were more effective in reversing the effect of Flt a-1. Transfection of HUVECs with 5nM siFlt-1 resulted in significant recovery of cell proliferation in the 2nM Flt a-1 condition (Fig 6B), while control siNEG had no effect. Thus, we concluded that suppression of cell proliferation by Flt a-1 was due to upregulation of sFlt-1.

### Histone modifications play a role in sFlt-1 expression

**Fig 7:**
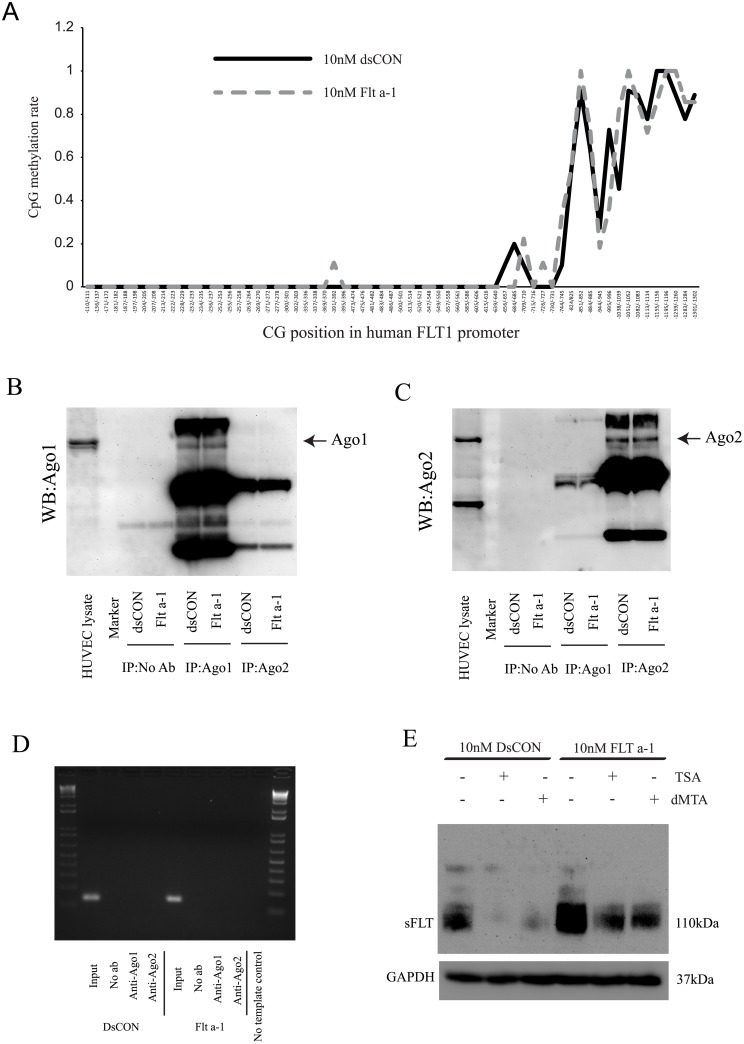
Histone modification may mediate Flt a-1 effects. (A) DNA methylation assay of flt-1 promoter. 65 CG sites (from -110/-111 to -1301/-1302) were analyzed by bisulfite sequencing. The difference between dsCON and Flt a-1 transfection was not confirmed. (B) Immunoprecipitation of Ago1. (C) Immunoprecipitation of Ago2. (D) ChIP assay of Ago1 and Ago2 on flt-1 promoter. Ago1 and Ago2 association to flt-1 promoter was not observed. (E) HUVECs transfected with Flt a-1 or dsCON were treated with an inhibitor of histone deacetyltransferase or histone methytransferase, which reduced basal and Flt a-1 induced sFlt-1 protein expression, indicating the relevance of histone modifications in upregulation of sFlt-1 by Flt a-1.

To elucidate the mechanism of sFlt-1 activation, we first tested whether Flt a-1 affects methylation levels of the flt-1 promoter by treating DNA of Flt a-1 or dsCON treated cells with bisulfite which converts cytosine to uracil, leaving 5-methylcytosine unaffected. Subsequent sequencing showed no significant difference between the two groups implying that Flt a-1 transfection doesn’t alter DNA methylation status of the flt-1 promoter (Fig 7A). Next, we tested if association of argonaute 1 or 2 (Ago1 or Ago2) with the flt-1 promoter was involved with sFlt-1 activation using a chromatin immunoprecipitation (ChIP) assay. Ago proteins play an essential role in RNA induced gene silencing by being recruited to the target gene by ncRNA and leading to mRNA cleavage or translational inhibition [28, 29]. Also, some studies showed that saRNAs guide Ago proteins to either promoter DNA or promoter transcripts and enhance gene expression [30–32]. However, we were not unable to detect Flt a-1 mediated induction of a purported association between the flt-1 promoter and Ago1 or Ago2 (Fig 7B–7D). Finally, to evaluate potential histone modifications in sFlt-1 activation, we treated Flt a-1 or dsCON transfected cells with trichostatin A (TSA), a histone deacetylase inhibitor, or with 5’-deoxy-5’-(methylthio) adenosine (dMTA), a histone methyltransferase inhibitor. Overnight treatment with TSA or dMTA significantly reduced both sFlt-1 basal expression and Flt a-1 induced sFlt-1 activation, suggesting that histone acetylation and methylation may both be required for sFlt-1 activation (Fig 7E).

## Discussion

Angiogenic homeostasis requires proper orchestration of proangiogenic and antiangiogenic factors. Perturbation of normal angiogenic control contributes to the pathology of numerous diseases including macular degeneration, diabetic retinopathy, benign and malignant tumors, and inflammatory diseases [4, 33]. sFlt-1 is an endogenous angiogenesis inhibitor that sequesters VEGF, a major angiogenesis inducer. sFlt-1 has been successfully used in antiangiogenic gene therapy through viral or nonviral delivery of sFlt-1 encoding DNA in animal models of ocular neovascularization and cancer [34, 35] as well as human clinical trials [12, 13]. In this study, we investigated a novel antiangiogenic strategy consisting of small activating RNA targeting the flt-1 promoter to enhance expression of sFlt-1.

Since Li et al. first noticed that promoter-targeting double stranded RNAs originally designed to silence gene expression actually resulted in activation of the targeted genes, multiple studies have tested the applicability of saRNA targeting on other genes including E-cadherin, p21, VEGF, progesterone receptor, p53, and Nanog [18, 20, 32, 36]. Although saRNA provides a tool for upregulation of specific genes and introduces a potential new role of ncRNAs in modulating gene expression, the molecular mechanism of saRNA is not fully understood.

To test the applicability of saRNA in inducing sFlt-1 expression, we designed three saRNA constructs, each targeting a different region of the flt-1 promotor. The location of the target sequence relative to the transcriptional start site (TSS) is one of the determining factors of a successful saRNA design. Some studies suggest targeting 200-1200bp upstream of the TSS is effective while others successfully used saRNAs that overlap the TSS [20, 21]. Yue et al. has even shown that duplex RNAs targeting downstream of the 3’ terminal region of the PR gene induced transcriptional activation [22]. In our study, we designed saRNAs targeting locations ranging from 724–1823bp upstream of the TSS. One of the three saRNAs targeting 724–742bp upstream of the TSS significantly increased sFlt-1 expression in RNA and protein levels. We demonstrated that sFlt-1 can be induced by saRNAs and that this activation is target sequence-dependent. While Flt a-1 transfection induced an increase of sFlt-1 at both mRNA and protein levels, it enhanced mRNA but not protein levels of mFlt-1. mRNA expression levels do not consistently correlate with protein abundance [37]. For example, Schwanhausser et al. found that approximately 40% of the variability in protein levels is explained by mRNA levels and that protein abundance seems to be controlled mainly by translation rate and to a lesser degree by protein stability to [38].

Several studies found that Ago proteins are required for RNA activation, as knockdown of Ago2 abolished RNA activation [14, 22, 24]. However, Janowski et al. (2007) did not detect increased localization of Ago 1 or Ago 2 to the promoter upon RNA activation of progesterone receptor. Similarly, we did not find flt-1 promoter association of Ago 1 or Ago 2 upon Flt a-1 activation of sFlt-1, suggesting that the involvement of Ago proteins is not a universal mechanism for RNA activation.

Epigenetic mechanisms such as DNA methylation and histone modification play important roles in the regulation of gene expression. DNA methylation has been shown to be one of the mechanisms employed by small interfering RNAs in plant and human cells [39–41], where saRNA reversal of methylation can activate gene expression [42]. However, we found that the flt-1 promoter in HUVECs is almost entirely unmethylated and that Flt a-1 did not induce any significant changes in the DNA methylation level. In contrast, our study suggests that histone modification may be a factor in Flt a-1 medicated gene activation, as both a histone deacetylase inhibitor (TSA) and a methyltransferase inhibitor (dMTA) significantly reduced basal and activated levels of sFlt-1 expression. Thus, deacetylation and methylation of histone proteins may be required for sFlt-1 activation by Flt a-1. Our future studies will further elaborate the mechanisms of RNA activation, and optimize saRNA delivery and targeting for the purpose of developing agents with long-acting therapeutic potential.

Our study demonstrates that saRNA can be successfully used to enhance sFlt-1 expression in a sequence specific manner, providing an additional tool to regulate angiogenesis via flt-1 promoter targeting. We showed that the sFlt-1 produced is fully functional, effectively inhibiting three key features of angiogenesis: endothelial cell proliferation, migration and tube formation. It also suggests there are a variety of epigenetic mechanisms through which different saRNAs activate their target genes. More broadly, saRNAs can provide a useful means for manipulation of gene expression in laboratory and therapeutic development. Determining how genes, cell types and epigenetic context influence the activity of individual saRNAs is a productive area for further study.

## Supporting information

S1 Fig Flt a-1 concentration-dependent mRNA expression of sFlt-1. mRNA levels of sFlt-1 at 72 hours after transfecting HUVECs with different concentrations of Flt a-1; 0, 1, 5, 10, or 30nM.
(EPS)
